# Aging Anxieties and Disturbed Eating in Female Students: It’s not all About Aging Appearance Concern

**DOI:** 10.5964/ejop.v14i1.1390

**Published:** 2018-03-12

**Authors:** Béré Mahoney

**Affiliations:** a Department of Psychology, University of Worcester, Worcester, United Kingdom; Department of Psychology, Webster University Geneva, Geneva, Switzerland; Edinburgh Napier University, Edinburgh, United Kingdom

**Keywords:** female students, disturbed eating, aging anxiety, psychological losses, interpersonal losses

## Abstract

Fear and anxiety about aging have increased amongst female university students and these personal aging concerns are associated with disturbed eating, also prevalent in this group. Evidence suggests concern about aging appearance could account for the link between aging anxiety and problem eating in young women due to their belief in the thin – youth ideal. However, whether appearance concern is the strongest aging anxiety predictor of global and specific disturbed eating behaviors is unclear. The study examines this in a sample of female students at a Midlands university in the United Kingdom (N = 200, 18 – 39 years) who completed the Anxiety about Aging Scale and the Eating Disorders Inventory-3. The findings show general and a model of four aging anxieties predicted significantly greater global disturbed eating with medium and large effects sizes respectively. However, greater anxiety about the psychological challenges and interpersonal losses associated with aging best predicted global and specific disturbed eating behaviors and aging appearance concern was a weaker predictor. Implications for interventions targeting female students eating behavior are considered.

Mental health problems are increasing amongst university students (Pedrelli, Nyer, Yeung, Zulauf, & Wilens, 2015; Royal College of Psychiatrists, 2011) with prevalence rates greater than in the general population (Bacigalupe, Esnaola, & Martin, 2016; Stallman, 2010; cf. Blanco et al., 2008). Given that more young adults are entering university (OECD, 2016) it is vital to identify student groups at greatest risk of mental health problems, their psychological difficulties and factors harming their psychological health (Seldon, 2015; Universities UK, 2015). Currently, females rather than males are one student group at greatest risk of mental health problems (Stallman, 2010) and this gender gap is very apparent for disturbed eating (DE) and diagnosable eating disorders (ED) (Pedrelli et al., 2015; YouGov, 2016).

ED and DE are more prevalent in women (Keski-Rahkonen & Mustelin, 2016; Makino, Tsuboi, & Dennerstein, 2004) particularly during late adolescence and early adulthood (Makino et al., 2004; Mental Health Foundation, 2016). However, women at university are especially at risk (Eisenberg, Nicklett, Roeder, & Kirz, 2011; Keski-Rahkonen & Mustelin, 2016; Power, 2016). A survey of 1061 university students in the United Kingdom found that over twice as many females (18%) than males (7%) described their mental health difficulty as an eating disorder (YouGov, 2016). Identifying factors fostering DE in female students is clearly important given problem eating is a precursor of ED (Mental Health Foundation, 2016) and the group’s DE is relatively stable during their university career (Berg, Frazier, & Sherr, 2009). Furthermore, the prognosis for women with ED is poor compared to other mental health problems and physical, psychological and social costs to sufferers are substantial (Pwc, 2015).

One factor that could be contributing to female students’ greater DE is their attitude to aging. Personal aging is a salient issue for young adults generally (Wohlmann, 2012). However, those at university are increasingly anxious about the transition to adulthood and are more negative in their attitudes to aging (Smith et al., 2017). Attitudes to aging, measured through such constructs as ageism, fear of aging and aging anxiety (Lynch, 2000; Smith et al., 2017) can protect and harm health (World Health Organisation, 2016). Positive attitudes are beneficial to health (Keyes & Westerhof, 2012) and those optimistic about aging engage in more health promoting behaviors such as being active physically and socially (Doyle, McKee, & Sherriff, 2012). Conversely, negative attitudes are linked to poor physical and psychological health (Brunton & Scott, 2015; Keyes & Westerhof, 2012; Swift, Abrams, Lamont, & Drury, 2017) including poorer emotional well-being in young women (Barrett & Toothman, 2016). Importantly, young women, defined broadly in studies as 18-39 year olds and including university students, are more negative about aging than their male peers as well as middle aged and older adults of both sexes (Barrett & von Rohr, 2008; Brunton & Scott, 2015; Lynch, 2000; Sargent-Cox, Rippon, & Burns, 2014). Young women are also more pessimistic about their aging appearance (Barrett & Robbins, 2008; Brunton & Scott, 2015; Gendron & Lydecker, 2016) and female university students negative about personal aging are more likely to ‘dread’ looking older (Chonody & Teater, 2016). Young women also stigmatize their future aging appearance in their discourse about the transition to middle and later life (Bybee, Merisca, & Wells, 1999; Bybee & Wells, 2002; Bybee & Wells, 2006). Furthermore, women in general with negative attitudes to aging report greater global DE, body dissatisfaction and drive for thinness (Barrett & Robbins, 2008; Becker, Diedrichs, Jankowski, & Werchan, 2013; Gendron & Lydecker, 2016; Robert-McComb & Massey-Stokes, 2014; Runfola et al., 2014). This is consistent with evidence that aging and DE have common appearance based determinants (Keel & Forney, 2013; Stice, Ng, & Shaw, 2010). Quantitative and qualitative studies also show concern about aging appearance and the body are central to women’s beliefs about aging and DE (Becker et al., 2013; Twigg, 2004). Thus, it is plausible that female students’ more negative attitudes to aging, and appearance concern particularly, could be contributing to their greater level of DE and poor adjustment to developmental transitions across adulthood (Smith et al., 2017). However, aging research often neglects the consequences of young adults concerns about aging for their health (Smith et al., 2017; Wohlmann, 2012).

Lasher and Faulkender’s (1993) multidimensional sociocultural theory of aging anxiety provides a possible theoretical framework explaining why aging concerns and DE are linked in women. Aging anxieties are concerns and fears about getting older (Lasher & Faulkender, 1993; Lynch, 2000). Lasher and Faulkender’s theory, developed using university student and community samples, differentiates these into four related but distinct dimensions measurable with the Anxiety about Aging Scale (AAS). The ‘fear of old people’ dimension reflects defensiveness about aging expressed through the avoidance of social interaction with the elderly; the ‘psychological concern’ and ‘appearance concern’ dimensions reflect anxieties about psychological challenges faced when adjusting to aging and associated appearance changes respectively; and, the ‘fear of losses’ dimension reflects concern about aging-related social and interpersonal losses. These anxieties are experienced across the lifespan and each has distinct psychological and social consequences for adjustment to aging and interactions with older people (Harris & Dollinger, 2003; Lasher & Faulkender, 1993). The meaning of these anxieties also varies between individuals (Lasher & Faulkender, 1993). Aging appearance concerns are likely to be important to women given that the ‘double jeopardy’ of ageism and sexism is more salient to them (Chrisler, 2011; Fineman 2014) and aging appearance concerns are alleged to motivate activities the individual believes will “...preserve the look of youth” (Lasher & Faulkender, 1993, p. 248). Similarly, research on problem eating shows the desire to appear young characterises DE and ED (Garner, Olmstead, & Polivy, 1983; Gupta, 1990). Measured with the Eating Disorders Inventory (EDI) maturity fears scale, these apprehensive thoughts focus on biological, psychological and social changes associated with physical maturity (Garner et al., 1983) and similarly motivate individuals’ to engage in DE behaviors they believe will achieve a slimmer physique and thus “experience themselves as younger” (Garner, 2004, p. 77). However, maturity fears are not considered widely in research on aging attitudes and DE despite their conceptual and functional similarities with aging appearance concerns. Both are appearance based, involve an aversion to maturity-related physical changes such as weight gain, entail the belief that a youthful physique is desirable and motivate behaviours believed to retain a youthful body. This is consistent with recent evidence that female students endorse a so-called ‘thin - youth ideal’ or the belief that ‘youthfulness’ is denoted by ‘slimness’, and this is associated with greater body dissatisfaction (Gendron & Lydecker, 2016). Also, the EDI maturity fears scale has been used to measure fear of aging amongst university students because of the apparent conceptual similarities of these measures (Smith et al., 2017). Thus, theoretically the link between aging anxiety and DE in women might be strongest for aging appearance concerns and DE behaviors but especially those problem eating behaviors related closely to belief in the thin – youth ideal; namely body dissatisfaction, drive for thinness and maturity fears.

Research findings suggest that women generally endorse the thin-youth ideal. They are more likely to engage in ‘body practices’ to achieve a slim physique they believe denotes youth and healthiness (Carter, 2016) and anti-aging bodywork as a strategy to delay or reverse appearance aging and avoid the stigma of ‘looking old’ (Chrisler, 2011; Muise & Desmarais, 2010). Coupland’s (2007) discourse analysis of lifestyle magazines and skincare advertisements confirms women face social pressure to “remedy” (p. 48) the ‘problem’ of facial aging with antiaging “practices” (p. 48) and for young women “…the onset of aging (is) a serious issue” (p. 48). Women also associate ‘old’ with ‘fat’ (Rubinstein & Foster, 2013; Runfola et al., 2014) and those engaging in ‘fat talk’ or speech promoting the thin ideal, also have greater aging appearance concern, greater global DE and drive for thinness (Becker et al., 2013). Women at university might be at particular risk of these experiences given attending university is a developmental transition that often triggers psychological problems in female students, including DE (Piontkowski, 2014; Royal College of Psychiatrists, 2011). Younger women also experience greater social pressure to attain the thin ideal (Pruis & Janowsky, 2010) and when worried about aging prefer dietary restraint and food monitoring as weight management strategies (O’Reilly, Thomlinson, & Castrey, 2003). Young women’s appearance concerns also focus on their body shape (Goodman, 1994) while women’s body dissatisfaction and ‘fat talk’ decline across adulthood (Becker et al., 2013; Field-Springer, 2012; Montemurro & Gillen, 2013). Female students with greater aging appearance concern also engage in more body surveillance activities indicating they define their bodies by how they look rather than feel (Gendron & Lydecker, 2016).

However, whether the link between aging anxiety and DE in young women is due largely to their high level of appearance concern is unclear from current evidence. Quantitative studies sometimes use bespoke single-item measures with unknown psychometric properties to assess aging appearance concern, such as Barrett and Robbins (2008) who asked women how frequently they worried about “being less attractive as a woman” as they became older; and, the AAS appearance concern subscale has been used by researcher in isolation from it’s other subscales preventing comparisons of how different aging anxieties associate with DE (Becker et al., 2013; Slevec & Tiggemann, 2010). Also, quantitative studies typically assess a narrow range of problem eating behaviors such as global DE and cardinal risk behaviors (Becker et al., 2013). For example, Gendron and Lydecker (2016) used the AAS in full but body objectification and body image avoidance measures rather than explicit measures of DE. Similarly, qualitative studies provide rich data on broad themes in women’s discourses around aging, appearance and problem eating behaviour but are less clear on which aging concerns are associated with specific DE behaviors (Becker et al., 2013). Thus, researchers have tended to assume or infer that aging appearance concern most strongly associates with DE *per se* despite research suggesting otherwise. For example, specific aging-related concerns associate with different psychological maladaptations including DE behaviors (Barrett & Robbins, 2008; Benton, Christopher, & Walter, 2007; Gendron & Lydecker, 2016). Furthermore, female university student’s satisfaction with their current life, self and physical appearance relate strongly to DE (Matthews, 2009) as do female students fear of their future appearance and health-related self (Lucette, 2012).

Therefore, this study aims to investigate more explicitly than current research the link between aging appearance concern and other aging anxieties, and global and specific DE behaviors in a sample of 18 – 39 year old women attending university. Unlike much of the previous research both general and different aging anxieties are measured with the AAS and used as two separate models to predict global and a broad range of DE behaviors, including body dissatisfaction, drive for thinness and maturity fears. The following hypotheses were tested: first, aging appearance concern will be significantly greater than other aging anxieties and the mean level of maturity fears is expected to fall within the typical clinical range that is considered as relatively high for nonclinical populations (Garner, 2004); second, the model of general aging anxiety will predict significantly more global and specific DE behaviors, with the strongest prediction and effect size for body dissatisfaction, drive for thinness and maturity fears; and, the model of Lasher and Faulkender’s (1993) four aging anxieties will also predict significantly more global and specific DE behaviors, with the strongest prediction and greater effect size also for body dissatisfaction, drive for thinness and maturity fears. In the second model appearance concern is expected to be the aging anxiety that best predicts global and specific DE behaviors, and especially body dissatisfaction, drive for thinness and maturity fears.

## Method

### Design

Simple (Model 1) and multiple (Model 2) linear regression models were used to test if aging anxieties predicted DE. The simple model used general aging anxiety (total scores on the AAS) to predict global DE (total scores on the Eating Disorders Inventory-3) (EDI-3) and specific DE behaviours (scores on the twelve EDI-3 scales). The multiple linear regression used scores on the four AAS subscales (appearance concern, fear of losses, fear of old people and psychological concern) entered simultaneously as the model to also predict global DE and twelve specific DE behaviours.

### Participants

A priori power analyses were run using G*Power 3.1.9.2 (Faul, Erdfelder, Buchner, & Lang, 2009) to identify the minimum sample sizes needed to test the simple and multiple linear regression models. Baseline equations with one (simple linear regression model) and four (multiple linear regression model) predictor variables were used with Cohen’s (1992) recommended convention of testing a medium effect size for ƒ^2^ (medium = .15) at power = .80 with α level < 0.05. The minimum sample sizes needed for the analyses were *N* = 55 with one predictor and *N* = 85 with four predictors. The final opportunity sample recruited of 200 women aged 18 – 39 years (*M* = 21, *SD* = 4.50) attending a Midlands university in the United Kingdom exceeded both the samples sizes required. Demographic information was collected on participant’s age (in years) and ethnicity (White, Black, Asian, Mixed or Other). Self-reported reported height and weight were also collected and they have good concordance with objective measures amongst university students (Quick et al., 2015). To screen for known eating disorders participants responded *yes* or *no* to the question “Do you currently or ever have been diagnosed with an eating disorder?”

### Materials

Lasher and Faulkender's (1993) Anxiety about Aging Scale (AAS) was used. This 20-item self-report scale has four subscales with good internal consistency (Lasher & Faulkender, 1993): fear of old people (Cronbach’s α = .78); psychological concern (Cronbach’s α = .74); appearance concern (Cronbach’s α = .71); and fear of losses (Cronbach’s α = .69). These are correlated but independent factors (Lasher & Faulkender, 1993) and responses to items are rated on a five point scale (5 = strongly agree to 1 = strongly disagree). High scores indicate greater anxiety and general aging anxiety is calculated by totalling subscale scores. Recent research confirms the AAS four factor structure, internal consistency (fear of old people Cronbach’s α = .80; psychological concern Cronbach’s *α* = .80; appearance concern Cronbach’s α = .73; fear of losses Cronbach’s α = .69), validity as a measure of aging anxieties in female and male adults aged 20 – 97 years and it’s generally age and gender invariant factorial structure (Sargent-Cox et al., 2014).

The EDI-3 (Garner, 2004) was also used. Clinicians and researchers use this 91-item self-report scale to measure psychological traits important to the development and maintenance of eating disorders. Its twelve scales also measure the presence of DE. Drive for thinness, body dissatisfaction and bulimia scales measure cardinal DE risk behaviors or weight preoccupation (Garner, 2004). Eight of the remaining nine scales form four strongly correlated pairs measuring domains of psychological maladjustment. Personal ineffectiveness is measured by low self-esteem and personal alienation scales; interpersonal problems are measured by interpersonal insecurity and interpersonal alienation scales; affective problems are measured by interoceptive deficits and emotion dysregulation scales; and, over control is measured by perfectionism and asceticism scales. The maturity fears scale is not part of these domains. Items are rated on a six-point scale ranging from ‘always’ to ‘never’, with the four most pathological responses in descending order weighted 4, 3, 2 and 1 with the two least pathological responses weighted 0. High scores indicate more DE and global DE is calculated by totalling scale scores. The EDI-3 is the most recent version of the Eating Disorder Inventory (EDI) (Garner et al., 1983) and has good internal consistency (Cronbach’s α = .80) (Garner, 2004), convergent, discriminant and external validity (Clausen, Rosenvige, Friborg, & Rokkedal, 2011; Garner, 2004). The EDI-3 consists of all 91 items from the earlier Eating Disorders Inventory-2 (EDI-2) but with scale revisions informed by contemporary eating pathology research (Garner, 2004). Detailed discussion of the revisions is beyond the scope of this paper (see Garner, 2004). However, relevant key changes are the use of nine rather than eight general psychological scales and reassignment of some EDI-2 items to different scales in the EDI-3 (e.g. items from the EDI-2 interoceptive awareness scale have been reassigned to the EDI-3 bulimia and body dissatisfaction scales) (Garner, 2004). Some psychological scales have also been renamed (e.g. EDI-2 impulse regulation scale is relabelled as emotion dysregulation in the EDI-3). The EDI-3 also contains new scales consisting of items drawn from EDI-2 scales (e.g. the EDI-3 interpersonal alienation scale consists of items taken from EDI-2 interpersonal distrust, impulse regulation and social insecurity scales) and responses are rated on a four-point rather than the three-point scale used in the EDI-2 to increase the response range (Garner, 2004).

### Procedure

Female undergraduates attending a first year introductory Psychology lecture were invited to take part in the study. Each was given a Participant Information Sheet and Consent Form to read at the start of the lecture. At the end of the lecture the researcher answered questions about the study and those not wishing to participate were permitted to leave. Participants then completed their Consent Form and a questionnaire whilst in the room. The questionnaire asked first for demographic information and whether they currently or ever had been diagnosed with an eating disorder, followed by the AAS and the EDI-3. The process from consent taking to questionnaire completion took 20 – 30 minutes.

The local Research Ethics Committee approved the research.

## Results

A small number of values were missing from the data collected but these never reduced the usable sample size below those required. Thus, the sample n was reduced in such instances rather than using data imputation and the resulting variable sample sizes shown in Tables 1 and 2 (*N* = 198 – 200) reflect these adjustments.

### Demographic Information, Body Mass Index and Eating Disorders

Table 1 shows the number and percentage of the sample in different age bands, ethnic groups, body mass index (BMI) categories and reporting currently having or ever being diagnosed with an eating disorder. BMI was calculated by dividing weight in kilograms (kg) by height in metres (m) and then dividing the answer by height again (National Health Service, 2016)

**Table 1:** Demographic Information, Body Mass Index and Eating Disorders

Characteristic	*N*	%
Age		
18 - 21	144	72
22 - 29	25	13
30 - 39	30	15
Ethnicity		
White	198	99
Black	0	0
Asian	2	1
Mixed	0	0
Other	0	0
Eating disorders		
Yes	0	0
No	198	100
Body mass index		
Underweight <18.5	28	14
Healthy 18.5-24.9	139	70
Overweight 25-29.9	16	8
Obese 30-39.9	16	8

The majority of the sample were 18 – 21 years of age (72%) and self-described their ethnicity as ‘white’ (99%). None of the sample reported currently or ever being diagnosed with an eating disorder and the majority (70%) had a BMI in the ‘healthy’ range (*M* = 22, *SD* = 5.0) (National Health Service, 2016). No further analysis was run using the data shown in Table 1 due to sub-group small sizes.

### Description of Variables

Total AAS scores ranged from very low to high (Range = 27 to 90). The mean of 53.80 (*SD* = 11.56) is slightly higher than that reported by Lasher and Faulkender (1993) for women aged up to 35 years and female students aged 18 – 25 (Gendron & Lydecker, 2016) but similar to that in other research with undergraduates (Allan & Johnson, 2008; Harris & Dollinger, 2001). Mean scores on the AAS subscales also ranged from very low to high. Fear of losses (Range = 5 to 25, *M* = 15.0, *SD* = 3.80) was slightly greater than anxiety about appearance concern (Range = 5 to 25, *M* = 14.30, *SD* = 4.20) followed by psychological concern (Range = 5 to 25, *M* = 12.86, *SD* = 3.70) and fear of old people (Range = 5 to 25, *M* = 11.70, *SD* = 3.40). A one-way analysis of variance was run to test if appearance concern was greater than the other aging anxieties measured. Mauchley’s test indicated the assumption of sphericity had been violated, χ^2^(5) = 24.40, *p* < .001 and degrees of freedom were corrected with Greenhouse-Geisser estimates of sphericity (ϵ = .92). The results show a significant effect of aging concern, *F*(2.78, 554.7) = 51.5, *p* < .001 and Bonferroni post hoc analysis shows scores on all AAS subscales significantly differed from one another except fear of losses compared to appearance concern. This provides some support for the prediction that appearance concern would be high in the sample but only compared to psychological concern and fear of old people.

Table 2 shows the mean scores on the EDI-3 with those in the typical clinical range (Garner, 2004) flagged.

**Table 2:** Mean Scores on the Eating Disorder Inventory-3 (EDI-3)

Scale	*N*	*M*	*SD*
EDI-3 total	198	95.70	49.60
Drive for thinness	200	9.25	7.56
Body dissatisfaction	200	17.44	10.40
Bulimia^a^	200	5.18	5.11
Low self-esteem	200	6.20	5.48
Personal alienation	198	6.43	5.44
Interpersonal insecurity^a^	198	6.62	5.30
Interpersonal alienation^a^	198	6.37	4.40
Interoceptive deficits	199	8.70	7.30
Emotional dysregulation^a^	198	5.90	4.64
Perfectionism^a^	200	9.72	5.60
Asceticism	198	5.11	4.40
Maturity fears^a^	200	8.70	5.80

^a^Mean scores in the typical clinical range (Garner, 2004).

Global DE scores (Range = 9 to 257) and scales scores (Range = 0 to 38) also ranged from very low to high. Six of the EDI-3 scale means were within the low, but six were within the typical clinical ranges although this is not unusual in nonclinical groups (Garner, 2004). As predicted, DE-related maturity fears were relatively high being one of six EDI-3 scale score means within typical clinical ranges (Garner, 2004). A one-way analysis of variance was run to explore whether levels of specific DE behaviors differed in the sample. Using standardised EDI-3 scale scores, Mauchley’s test indicated the assumption of sphericity had been violated, χ*^2^*(65) = 439.30, *p <* .001 and degrees of freedom were corrected with Greenhouse-Geisser estimates of sphericity (ϵ = .72). The results show scores on the maturity fears scales were not significantly higher than other scale scores and none of the EDI-3 scale scores differed significantly from one another, *F*(7.93, 1562) = .008, *p* = 1.00.

### Regression Analyses

Simple and multiple linear regression models of AAS total scores and the four AAS subscale scores respectively were used to predict EDI-3 total and scale scores. To reduce the likelihood of Type I errors a Bonferroni Correction was applied for each wave of analysis (.05/13 = *p* < .003). The data met diagnostic criteria for running regression analyses. No standardised residuals exceeded the critical value of 3 and less than 1% had a value > 2.5 indicating the data met the assumption of normality. VIF test values were < 2.5 and Durbin-Watson test values were neither >3 nor <1 indicating the data met the criteria for the absence of multicolinearity and the independence of errors respectively.

The results of the simple regression analyses are in Table 3.

**Table 3:** Results of Regression Model Using Total Anxiety About Aging Scale (AAS) Scores to Predict Eating Disorder Inventory-3 (EDI-3) Total and Subscale Scores

Variable	EDI-3	DT	BD	B	LSE	PA	II	IA	ID	ED	P	A	MF
Constant^a^	-35.80	-6.92	-4.78	-3.47	-6.78	-6.25	-3.58	-1.24	-6.64	-3.43	8.45	-2.31	1.45
AAS^a^	2.44*	0.30*	0.41*	0.16*	0.24*	0.24*	0.19*	0.14*	0.29*	0.17*	0.02	0.14*	0.14*
*R*^2^	.32	.21	.21	.13	.25	.24	.17	.14	.20	.18	.00	.13	.07
*F*	91.78*	51.50*	51.35*	29.33*	66.90*	64.10*	39.45*	30.65*	49.14*	43.97*	.47	29.00*	15.00*

*Note*. *R^2^* effect sizes: small = .10, medium = .30 and large = .50 (Cohen, 1992); AAS = Total Aging Anxiety Scale scores; EDI-3 = Eating Disorders Inventory – 3 total score; DT = drive for thinness; BD = body dissatisfaction; B = bulimia; LSE = low self-esteem; PA = personal alienation; II = interpersonal insecurity; IA = interpersonal alienation; ID = interoceptive deficits; ED = emotion dysregulation; P = perfectionism; A = asceticism; MF = maturity fears.

^a^Unstandardised beta coefficients.

*p < .003.

The results support the hypothesis that greater general aging anxiety (Model 1) will significantly predict more DE, accounting for 32% of the variance in global DE with *R^2^* = .32 considered as a medium effect size using Cohen’s convention for simple linear regressions (1992). The hypothesis that general aging anxiety will also account for significant variance and higher scores on the EDI-3 scales was supported with the exception of perfectionism. The effect sizes were medium (Cohen, 1992) for drive for thinness, body dissatisfaction, low self-esteem, personal alienation, interpersonal insecurity, interoceptive deficits and emotion dysregulation (*R^2^* = .17 – .25) although small (Cohen, 1992) for bulimia, interpersonal alienation, asceticism and maturity fears (*R^2^* = .07 – .14). Thus, there was some support for the hypothesis that general aging anxiety will predict strongly body dissatisfaction and drive for thinness but not for maturity fears.

The results of the multiple regression analyses (Model 2) using AAS subscale scores as a model to predict EDI-3 total and scale scores are in Table 4.

**Table 4:** Results of Regression Model Using Anxiety About Aging Scale (AAS) Dimension Scores to Predict Eating Disorder Inventory-3 (EDI-3) Total and Subscale Scores

Variable	EDI-3	DT	BD	B	LSE	PA	II	IA	ID	ED	P	A	MF
Constant^a^	-32.34	-7.74	-4.57	-2.78	-6.18	-6.10	-3.55	-1.00	-5.23	-3.22	7.68	-2.31	2.71
Fear of old people^a^	0.06	0.30	0.22	-0.10	0.06	0.07	0.10	-0.10	-0.06	-0.01	-0.05	-0.03	-0.30
Psychological Concerns^a^	3.16*	-0.10	0.20	0.21	0.56*	0.49*	.24	0.24	0.69*	0.11	-0.14	0.18	0.41*
Physical Appearance^a^	1.83	0.41	0.51	0.14	0.10	-0.03	0.04	0.05	0.01	0.27*	0.02	0.11	0.18
Fear of Losses^a^	4.05*	0.59*	0.64	0.29	0.21	0.39*	.36*	0.32*	0.38	0.27	0.27	0.26	0.10
*R^2^*	.35	.23	.21	.15	.29	.29	.18	.19	.24	.21	.03	.16	.14
*F*	26.10*	14.63*	12.89*	8.72*	19.69*	20.10*	10.58*	11.40*	14.89*	12.85*	1.24	9.16*	8.00*
Cohen’s ƒ^2^	.54	.30	.30	.20	.40	.40	.20	.20	.30	.30	.03	.20	.20

*Note.* Cohen’s ƒ^2^ effect sizes: small effect size = .02, medium effect size = .15 and large = .35 (Cohen, 1992); EDI-3 = Eating Disorders Inventory – 3 total score; DT = drive for thinness; BD = body dissatisfaction; B = bulimia; LSE = low self-esteem; PA = personal alienation; II = interpersonal insecurity; IA = interpersonal alienation; ID = interoceptive deficits; ED = emotion dysregulation; P = perfectionism; A = asceticism; MF = maturity fears.

^a^Unstandardised beta coefficients.

*p ≤ .003.

The results support the prediction that Lasher and Faulkender’s (1993) model of four aging anxiety dimensions will account for significant variance in global DE (35%) with a large effect size (Cohen’s ƒ^2^ = .35) (Cohen, 1992). The results also support the hypothesis that the model will account for significant variance in specific DE behaviors with small to medium effect sizes (Cohen’s ƒ^2^ = .02 - .15) (Cohen, 1992). However, there was no support for the hypotheses that the model and aging appearance concern will best predict body dissatisfaction, drive for thinness and maturity fears. Fear of losses and psychological concern significantly predicted greater global DE but appearance concern and fear of old people do not and only three of the four aging anxiety dimensions were significant individual predictors of higher scores on some EDI-3 scales. Greater psychological concern predicted lower self-esteem and greater personal alienation that combined indicate personal ineffectiveness, as well as interoceptive deficits and maturity fears. Greater fear of losses also predicted greater personal alienation as well as greater drive for thinness and more interpersonal insecurity and interpersonal alienation that combined indicate greater interpersonal problems. Appearance concern predicted only greater emotional dysregulation while fear of old people predicted no EDI-3 scale scores.

## Discussion

The study investigated whether general and different aging anxieties predicted global and specific DE behaviors in a sample of female university students. The results are broadly consistent with research showing their aging appearance concerns and maturity fears are high (Brunton & Scott, 2015; Gendron & Lydecker, 2016; Smith et al., 2017). However, results were less consistent with evidence that suggests female students appearance concerns drive the link between their aging anxiety and DE (Becker et al., 2013; Gendron & Lydecker, 2016) and that their belief in the thin – youth ideal would lead their aging anxieties and appearance concern particularly to be strong predictors of DE behaviors closely linked to this (i.e. body dissatisfaction, drive for thinness and maturity fears) (Gendron & Lydecker, 2016).

The high level of appearance concern (cf. Gendron & Lydecker, 2016) and clinically typical level of maturity fears (Garner, 2004) found are consistent with other studies showing that not only do young women fear aging but that looking ‘old’ and thus less physically attractive is an important part of these fears (Barrett & Robbins, 2008; Bybee & Wells, 2006). However, different to other studies (Gendron & Lydecker, 2016) aging–related social and interpersonal losses in addition psychological challenges faced adjusting to aging were equally or more anxiety provoking for the female students sampled than appearance concerns. This suggests that their personal aging concerns are not exclusively appearance based and confirms research findings suggesting students generally are increasingly anxious about developmental transitions *per se* (Smith et al., 2017) and that young women negative about aging have poorer emotional well-being (Barrett & Toothman, 2016).

Despite some dissimilarities with other studies, the study findings suggest clearly that the greater aging anxiety reported by female students could be contributing to their high levels of DE. The models using general aging anxiety (Model 1) and Lasher and Faulkender’s (1993) four aging anxieties (Model 2) produced similar results consistent with those in other studies showing negative attitudes to personal aging harm eating behaviors of women generally (Barrett & Robbins, 2008; Becker et al., 2013). However, using Lasher and Faulkender’s (1993) model of four aging anxieties rather than general aging anxiety as a single predictor has value as a framework for describing how different aging anxieties associate with DE in the female students sampled given different aging anxieties predicted relatively discrete DE behaviors. This is consistent with available research (Barrett & Robbins, 2008; Becker et al., 2013; Gendron & Lydecker, 2016) that younger women concerned about aging report they would engage in potentially maladaptive eating behaviors to control their weight (O’Reilly et al., 2003), sociocultural models of DE that identify psychosocial variables as risk factors (Keel & Forney, 2013; Stice et al., 2010) and evidence that different aging anxieties predict different aspects of DE (Gendron & Lydecker, 2016). Worryingly, the findings show elevated general aging anxiety predicts almost all psychological maladjustments measured by the EDI-3. This extends previous observations (Becker et al., 2013; Gendron & Lydecker, 2016) and suggests the adverse psychological consequences of aging anxiety for DE amongst female university students are more pervasive than available evidence indicates. This also confirms that even amongst women yet to reach later life and without a history of ED, having elevated concerns about personal aging is linked to a range of potentially maladaptive psychological responses in the form of most aspects of DE (Harris & Dollinger, 2003; Lasher & Faulkender, 1993). An additional implication is using cardinal risk DE behaviors (i.e. drive for thinness, body dissatisfaction and bulimia) as the sole measures of DE could underestimate the impact of aging anxieties on more psychological aspects of DE in this group.

Support for the hypotheses that aging anxiety would predict body dissatisfaction, drive for thinness and maturity fears more strongly than other DE behaviors because they are linked closely to the thin–youth was partial. Both aging anxiety models tested significantly predicted these specific DE behaviors, consistent with Gendron and Lydecker’s (2016) finding that amongst female students all the AAS subscales predict aspects of body consciousness that is linked to body dissatisfaction. However, both models predicted DE – related low self-esteem and personal alienation more strongly and this is consistent with evidence that students generally report high levels of symptoms linked to these psychological characteristics such as anxiety, depression and stress (Beiter et al., 2015). Unlike other studies of female students (Gendron & Lydecker, 2016) the link between aging appearance concern was weak although similar to others, specific aging anxieties predicted different DE behaviors and this confirms that different aging concerns associate with discrete psychological maladjustments (Barrett & Robbins, 2008; Benton et al., 2007) and that all aging anxieties measured by the AAS are important for different aspects of body dissatisfaction (Gendron & Lydecker, 2016). Similarly, the inconsistent link between aging anxiety and maturity fears is notable. These constructs allegedly foster behaviours that are ‘youth preserving’ (Lasher & Faulkender, 1993) and lead individuals to “experience themselves as younger” (Garner, 2004, p. 77) respectively. However, general aging anxiety’s effect size for maturity fears was small. This supports Smith et al.’s (2017) argument that this EDI scale measures more than young adults’ fear of aging and transition to later life. Yet, anxiety about the psychological challenges associated with aging did predict maturity fears. This suggests the latter has some value as a measure of fears about the transition to later life, but ones that relate to its psychological challenges. Furthermore, it suggests that Lasher and Faulkender’s (1993) argument that aging anxieties lead to ‘youth preserving’ activities that can be maladaptive requires further exploration.

Importantly, psychological concern and fear of losses best predicted both global and almost all other DE behaviors. This challenges the emphasis in the research on aging appearance concern as most important for DE generally (Becker et al., 2013; Rubinstein & Foster, 2013) and the finding that elevated concern about the loss of physical attractiveness is the aging concern associated most strongly with greater psychological maladjustment in younger women (Barrett & Robbins, 2008). It is possible that the limited predictive value of aging appearance concern for DE found could be an artefact of this AAS subscale. Its items assess feelings of dread if perceived as ‘old’ and lying to conceal one’s age. Thus, it could measure dysphoric emotions connected with appearance concern rather than body shape concerns *per se*. Appearance concern being the only aging anxiety dimension that predicted DE emotional dysregulation that indicates mood instability, impulsivity and anger (Garner, 2004) also suggests this. Interestingly, Gendron and Lydecker (2016) found greater appearance concern amongst female students only predicted the surveillance aspect of objectified body consciousness that indicates a focus on how the body ‘looks’ rather than feels, but did not predict body consciousness shame, control and body image avoidance. It is possible that aging appearance concern is a relatively discrete measure of a heightened focus on the ‘look’ of aging and the negative emotions attached to this (Becker et al., 2013). Furthermore, research on body image and health in adult women often uses mistakenly body dissatisfaction as a measure of DE (Kilpela, Becker, Wesley, & Stewart, 2015). The importance of conceptual relevance for fostering links between aging anxiety and DE might also explain why psychological challenges and not aging appearance concern significantly predicted maturity fears. Although the maturity fears scale measures fear of biological, psychological and social phenomena associated with physical maturity, these apprehensions entail beliefs about how psychologically challenging adjustment to these changes could be and not merely dysphoric emotions connected with appearance concerns (Garner, 2004). Similarly, fear of old people, that Lasher and Faulkender (1993) claim is not a direct measure of concern about one’s own aging, failed to predict global and any specific DE behaviors measured. This is inconsistent with research showing greater fear of old people predicts the control aspect of objectified body consciousness or the belief that one can and is responsible for controlling one’s appearance (Gendron & Lydecker, 2016). It is possible that fear of old people failed to predict any measure of DE used in the study because it reflects how individuals control their behaviour (i.e. avoidance of older people). Thus, fear of old could better predict other control based thoughts as found by Gendron and Lydecker (2016) rather than the measures of ‘over control’ in the EDI-3 (i.e. the perfectionism and asceticism scales, Garner, 2004) that are less explicit measures of control beliefs. Appearance concern could also have been less intense than other aging concerns and hence a poor predictor. However, this is unlikely given its mean intensity in the sample used was comparable to, if not greater than, other aging concerns measured, confirming its salience for young women (Barrett & Robbins, 2008; McConatha, Hayta, Rieser-Danner, McConatha, & Polat, 2004; Montemurro & Gillen, 2013; Yun & Lachman, 2006). Nevertheless, the findings suggest the link between aging anxiety and DE in this group is not all about aging appearance concern.

The predictive value of anxiety about the psychological challenges and interpersonal losses of aging for specific non-cardinal DE behaviours also confirms that dissatisfactions with different life domains relate to specific DE behaviors in female students (Matthews, Zullig, Ward, Horn, & Huebner, 2012) and that changes in women’s self-image, esteem and worth relate to different DE behaviors across adulthood (Landa & Bybee, 2007). For example, anxiety about the psychological challenges of adjusting to aging predict greater disturbance on EDI-3 scales that similarly measure concern about one’s ability to cope with psychological challenges. Namely, low self-esteem, an indicator of negative self-evaluation and the belief that one is unable to achieve one’s own personal standards; and, personal alienation that indicates emotional emptiness, aloneness, a desire to be someone else and a sense of separateness from others (Garner, 2004). Similarly, anxiety about aging’s social and interpersonal losses predict greater disturbance on EDI-3 scales that measure concern about social relationships. Namely, interpersonal ineffectiveness, an indicator of discomfort in social situations; and, interpersonal alienation an indicator of attachment impairments. These losses also predicted personal alienation (Garner, 2004) as well as greater drive for thinness, an indicator of belief in the socially prescribed notion of the thin ideal (Keel & Forney, 2013; Thompson & Stice, 2001). Crucially, personal alienation is the only DE psychological maladjustment predicted by multiple aging anxieties and these all entail concern about negative evaluation by others, low self-worth and anxiety about physical as well as social appearance. This is unsurprising given fear of negative evaluation is fundamental to DE (Gilbert & Meyer, 2005), aging anxiety and psychological maladjustment more generally (Bellew, Gilbert, Mills, McEwan, & Gale, 2006; Lundgren, Anderson, & Thompson, 2004). Aging anxiety and DE are themselves linked to maladaptive personality traits (Allan, Johnson, & Emerson, 2014; Cassin & von Ranson, 2005; Gao, 2009; Garner, 2004; Harris & Dollinger, 2003; Keel & Forney, 2013). This also suggests more general psychological maladjustments related to social appearance and self-esteem could have a role to play in their co-occurrence and qualitative research suggests health professionals delivering interventions enhancing body image view body dissatisfaction as an indicator of “... a greater sense of self-esteem problems that manifests through body image dissatisfaction” (Moulding & Hepworth, 2001, p. 313).

The findings also suggest that aging anxieties could associate with DE indirectly through psychosocial variables known to increase problematic eating. For example, anxiety about the psychological challenges and social and interpersonal losses associated with aging could make current and future life dissatisfactions salient. The link between these aging concerns and scores on EDI-3 scales that indicate personal and interpersonal ineffectiveness, along with personal alienation in particular, also suggest this and confirm research findings that DE amongst female university students associates with greater interpersonal problems (Stapleton & Empson, 2013) and alienation (Power & Lazenbatt, 2015). Those with less purpose in life also report more DE (Cottingham, Davis, Craycraft, Keiper, & Abernethy, 2014). Similarly, different life dissatisfactions relate to specific DE behaviors in this group (Lucette, 2012; Matthews, 2009; Matthews et al., 2012). Therefore, aging anxiety psychological concern and fear of losses could shape the content and frequency of negative thoughts about current and future social and physical appearance, phenomena linked to low self-worth and susceptibility to DE (Verplanken & Tangelder, 2011) and future research could explore the links between women’s aging anxieties, DE and other experiences known to influence these thoughts including pregnancy, the menopause, illness and bereavement (Kilpela et al., 2015).

Nevertheless, these interpretations are tentative given the study’s limitations. It focuses on female university students aged 18 – 39 years and future research could test for age differences in the link between different aging anxieties and specific DE behaviors in adult women given research shows these phenomena themselves change across the lifespan (Barrett & von Rohr, 2008; Montemurro & Gillen, 2013). Although at greater risk of DE and anxiety about their own aging, extrapolating its findings to young women not attending university is tentative and characteristics within the sample that could have influenced their appearance based concerns such as marital and parental status were not assessed but are important to consider in future research (Kilpela et al., 2015). Some mean EDI-3 scale scores were also within the typical clinical range. However, aging anxieties predicted DE behaviours both within and below typical clinical ranges with no significant differences between any EDI-3 scale scores. Furthermore, the reciprocal relationship between general psychological maladjustments and DE (Garner, 2004) make it unclear whether aging anxieties initiate or arise because of DE and future studies using longitudinal designs could be valuable for exploring this causal relationship and whether previously having an ED shapes this relationship.

Despite these caveats, the findings suggest holding negative beliefs about aging can have adverse consequences for current psychological health in the form of DE amongst women before they reach later life. Given nearly half the population of females within the 18-39 age range in developed nations are now entering university or other tertiary education institutions (OECD, 2016) the findings suggest a need for further research on the aging anxieties of this group and their health consequences. The findings also have specific implications for the prevention of DE in female students similar to those sampled in this study. First, aging anxiety is another psychosocial risk factor for DE amongst female university students with aging concerns predicting global, cardinal and almost all DE psychological maladaptations. Given effective interventions for reducing DE focus on altering attitudes and beliefs that foster DE (Stice et al., 2010), university-based interventions could benefit from adding anxiety-provoking beliefs about personal aging to those they challenge and beliefs about the psychological challenges and interpersonal losses associated with aging appear most important. Furthermore, the study shows aging anxiety predicts greater DE in a sample that excludes female university students with a known ED. This suggests aging anxiety might be another indicator of DE risk and ultimately ED in preventative work. This is consistent with current research which suggests that effective DE prevention programmes need to “zoom out” to consider more universal sociocultural factors that can encourage eating pathology (Bell, Rodgers, & Paxton, 2017, p. 89). Second, the findings signify female university students experience what could be a life transition double jeopardy that fosters DE: the transition to university triggers DE in this group whilst their concurrent anticipation of the future transition to later life, if sufficiently anxiety provoking, also worsens their DE. Third, current university-based approaches to tackling DE in students use measurable individual differences to identify those at greatest risk of DE (Musiat et al., 2014) and aging anxiety is another measurable construct that could be used for such a purpose. In particular, concern about the psychological challenges and interpersonal losses they could experience in later life best predict DE with personal alienation a correlate common to both. This confirms other research on female university students showing interpersonal concerns such as feelings of alienation and isolation associate strongly with DE in this group (Power & Lazenbatt, 2015). Finally, the findings challenge empirical and theoretical evidence suggesting the link between aging anxiety and DE is driven by aging appearance concerns. In contrast, they indicate the link is not all about appearance concerns. Consequently, interventions for preventing and reducing DE in female university students should focus on their broad psychological health issues, and particularly their beliefs about the challenges and losses they could face in later life.

Including aging anxieties as additional risk factors when screening for DE in female students and in the content of campus-based psychoeducational DE and ED prevention programmes is consistent with the current mental wellness agenda in universities (Universities UK, 2015). The apparent importance to DE of aging anxieties relating to broader social losses and psychological challenges in female students also suggest education around these aging beliefs could be useful to include in DE prevention programmes amongst students. Such programmes are more effective when they focus on promoting general wellness, resilience and coping with developmental challenges (Power, 2016), target those with sub - threshold ED and focus on specific DE risk factors rather than DE and ED in their content (Ciao, Loth, & Neumark-Sztainer, 2014) and aging anxieties have some synergy with these qualities.
